# Soldier, Items

**Published:** 1864-11

**Authors:** 


### SOLDIER ITEMS.

Stimulants and Tonics.—Chew a pinch or two of green tea when exhausted, or when on guard, or when on special hard duty, and repeat every half hour, more or less ; it enlivens without the subsequent debility of spirits. *Cayenne Pepper*, called "capsicum," acts similarly; a pinch at a time will modify that excessive weariness or sleepiness, and is far more powerful than tea, in all its good effects ; while its convenience for carriage in point of bulk, renders it the most valuable substance that a soldier can carry, as to nourishment, thirst, or invigorating powers. A single pinch in a cup of "flat" water will make it quite palatable. A third of a tea-spoonful taken at meals, morning, noon, and night, with the food or drink, not only invigorates digestion, but is a great antagonizer of dyspeptic and all bowel complaints in armies.

Stockings.—The feet will be blistered by a six hours' march in cotton stockings ; wear woolen, rubbing the soles with tallow or soap, if you can, when a heavy march is in prospect.

Food.—One pound of sugar mixed with three pounds of ground wheat or corn (with the bran) called "*Pinole*," is one of the most nutricious and healthy articles of food in the world for an army, and is easily carried. Jerked beef is next, made by cutting fresh beef in strips, and drying them in the sun, with as little salt as possible ; it will keep good a year.

Army Beverages.—Col. Dawes, an experienced East-Indian officer, says that coffee and tea should take the place of liquors, and that every man should have some as soon as he gets up in the morning, and also at sun-down. During the Crimean war, it was found that when the soldiers obtained warm coffee, they sustained fatigue and were comparatively healthy ; but when they were in the trenches, and could not get warm tea or coffee, they were subject to dysentery or bloody flux.

Swallowing Poison.—Stir in a glass of water a heaping teaspoonful each of salt and kitchen mustard, and drink it instantly—this will empty the stomach in a minute. To antagonize any poison that may be left, swallow the whites of two or three eggs ; then drink a cup or two of very strong coffee, or as much sweet milk or cream, if impossible to get coffee.

Poisoned Vines.—Apply a paste made of gunpowder, or sulphur, with milk ; renew night and morning, until cured. Live on gruel, soups, rice, and other mild food, having the bowels to act twice a day.

Signs of Death.—Bury no man unless his head is off, or the abdomen begins to turn green or dark, the only sure signs, but always sure, of actual death.

To Stop Bleeding.—Four or five drops of Perchlorid of Iron will check completely the flow of blood from all except the largest arteries; half a teaspoonful will arrest even their bleeding. Each non-commissioned officer should have two ounces of this in a flat tin bottle, wound around with a little cotton batting, on a bit of which the liquid could be dropped for application.

OBEDIENCE is not servility, it is a high duty ; it is not cowardly, but proudly honorable in a soldier. If your officer speaks sharply, it is neither to insult nor to browbeat ; it is to wake up attention, instant and implicit.

For every wounded soldier taken to the hospital in the Crimean war, twelve were taken on account of disease ; disease which could be avoided in more than half the cases by such care as the soldier can take of himself, as directed in these pages. Of the 15,000 lives lost in the Mexican war, only 1548 were from battle. The United States Sanative Commission report that 104 soldiers became sick to each 1000 in the present war.

SHIRTS.—A distinguished British Army Surgeon says : More than one half of all army diseases in warm countries are owing to the exposure of the abdomen to changes of temperature. Shirts should reach the thigh.

INNER CLOTHING.—Every garment which touches a soldier's skin should be woolen in all seasons, most important in the warmest weather. It is impossible to over-estimate the value of this one item to the health of an army.

LIMESTONE-WATER.—One teaspoonful of vinegar, in a pint of such water, will antagonize all its ill effects on the bowels of those unaccustomed to it.

DIRTY WATER.—As much powdered alum as will rest on a dime, stirred in a pail of water, will clarify it in five minutes.

SAVING LIFE.—In the first seven months of the Crimean campaign, the soldiers died at the rate of 60 out of a 100 per annum, while for the last five months of the war not so many soldiers died of disease as at home, owing to a more systematic and rigid attention to five things: 1st. Selecting healthful camps ; 2d. Enforcing strict cleanliness ; 3d. Avoiding *unnecessary* exposure ; 4th. Proper preparation of healthful food ; 5th. Judicious nursing.

A TRUE SOLDIER is considered one of the highest types of a man. But that officer merits not the name or the title he bears, who does not make the comfort and health of his men a subject of unceasing thought, and of the most indefatigable effort.

CAMP-GROUNDS.—An elevation is a hundred-fold better than a flat or a hollow ; open ground better than among trees ; better for health, safer from surprise, and stronger for attack and defense, even if it is calculated to stay but a few hours. Let the tent face the south, the top screned with brushwood, and if practicable with a floor of boards three inches above the ground, and a ditch around the tent six or eight inches deep.

DRINKING WATER improperly has killed thousands of soldiers. If possible avoid drinking anything on a march. If you must drink, the colder the water the less will it satisfy thirst. ☞ Half a glass of water drank in sips, swallowing each sip, with a few seconds interval, will more effectually satisfy thirst, and that without any danger, than a quart taken in the usual manner at one draught. It is greatly safest, *while marching*, to rinse the mouth only, but do that to the utmost extent desired, spirting out the water as soon as it becomes warm. Chewing even a stick or pebble moderates thirst.

Mittens, for cold weather, should have a thumb and one finger, the other three fingers together, so as to use the trigger handily.

Bowel Affections are said to be cured, if at all curable, by drinking from one half to four half pints of a tea made of the inner bark of the sweet-gum tree, boiled until of the taste and color of strong coffee, with or without sugar, cold or hot. The tree abounds southward.

Cromwell's Discharged Soldiers.—Immorality and irreligion are among the great evils of war. Knowing this, every Christian should be most diligent,not only in prayer for the soldiers,and in furnishing them with religious privileges in the camp, but in cherishing a strong and enlightened public religious sentiment. Public sentiment is a powerful stimulant to moral principle, as well as to patriotic feeling. It hence becomes the whole Christian community to frown upon Sabbath-day parades and displays. A country sometimes suffers immensely after a war is over, from the murders, robberies, thefts, and other depredations and immoralities of its own discharged soldiers. The principles and habits of the camp follow, or rather accompany, the men through life. In this aspect of the case, it becomes not only Christians who feel for men's immortal welfare, but it becoms all who have personal interests at stake, all who have properties or families to preserve, to see to the character of the camp. Cromwell kept up religion in his army. He had chaplains, prayers, Sabbaths, preaching, Bibles, psalm-books, and withal the bravest men that ever went into battle. And after their return to private life, history, in recording their heroic deeds, bears this testimony to their moral worth : Fifty thousand men, accustomed to the profession of arms, were at once thrown on the world. In a few months there remained not a track indicating that the most formidable army in the world had been absorbed into the mass of the community. The royalists themselves confessed that in every department of honest industry, the discarded warrior prospered beyond other men, that none was charged with any theft or robbery, that none was heard to ask an alms, and that, if a baker, a mason, or a wagoner attracted notice by his diligence and sobriety, he was in all probability one of Oliver's old soldiers.

Observance of the Sabbath.—*General Order No.* 7.—The Major-General Commanding desires and requests that in future there may be a more perfect respect for the Sabbath on the part of his command. We are fighting in a holy cause, and should endeavor to deserve the benign favor of the Creator. Unless in a case of an attack by the enemy, or some other extreme military necessity, it is commended to commanding officers that all work shall be suspended on the Sabbath ; that no unnecessary movements shall be made on that day; that the men shall, as far as possible, be permitted to rest from their labors ; that they shall attend divine service after the customary Sunday morning inspections, and that officers and men alike use their influence to insure the utmost decorum and quiet on that day. The General commanding regards this as no idle form. One day's rest in seven is necessary for man and animals. More than this, the observance of the holy day of the God of mercy and of battles is our sacred duty.

Washington, Sept. 6th, 1861.

